# CD74 facilitates immunotherapy response by shaping the tumor microenvironment of hepatocellular carcinoma

**DOI:** 10.1186/s10020-024-00884-x

**Published:** 2024-08-08

**Authors:** Jianghong Cheng, Junyang Li, Xinjie Jiang, Xi Ma, Bixuan Li, Han Zhai, Xianyang Luo, Yi Zhou, Junhua Wu, Zhiming Zhang, Shuai Chen, Yang Wang

**Affiliations:** 1 Xi’an Key Laboratory of Pathogenic Microorganism and Tumor Immunity, Xi’an Medical University, Xi’an, 710021 China; 2 Department of Otolaryngology-Head and Neck Surgery, School of Medicine, The First Affiliated Hospital of Xiamen University, Xiamen University, Xiamen, 361003 P.R. China; 3 Xiamen Key Laboratory of Otolaryngology Head and Neck Surgery, Xiamen, 361003 China; 4 Department of Breast Surgery, School of Medicine, The First Affiliated Hospital of Xiamen University, Xiamen University, Xiamen, 361003 P.R. China; 5 Teaching Hospital of Fujian Medical University, Fuzhou, Fujian 350004 P.R. China

**Keywords:** Hepatocellular carcinoma, CD74, Myeloid cells, Immune infiltration, IL-1B^+^ macrophages

## Abstract

**Background:**

CD74 is ectopically expressed in many tumors and can regulate tumor immunity. However, there are many gaps in the study of the prognostic value of CD74 expression and immune infiltration in hepatocellular carcinoma (HCC).

**Methods:**

An online tumor database was searched to obtain data on gene/protein expression. Immune infiltration analysis was performed using the Tumor Immune Estimation Resource and Comprehensive Analysis on Multi-Omics of Immunotherapy in Pan-cancer databases. Single-cell data were obtained from the Tissue-specific Gene Expression and Regulation, Single-cell Transcriptomes of Tumor Immune Microenvironment and Tumor Immune Single-cell Hub 2 databases.

**Results:**

CD74 was highly expressed in HCC patients. HCC patients with high CD74 expression who consumed alcohol or were negative for hepatitis virus had a better prognosis than patients with low CD74 expression. CD74 was mainly enriched in immune response regulation pathways. Both copy number variations in CD74 and CD74 expression patterns affected the infiltration levels of immune cells. Interestingly, CD74 regulated the differentiation of myeloid cells. CD74 in macrophages and dendritic cells (DCs) forms complex networks with malignant cells and hepatic progenitor cell (HPC)-like cells, respectively. High CD74 expression in HPC-like cells and malignant cells significantly decreased the fraction of C-type lectin domain family 9 A (CLEC9A)-cDC1^+^ DCs and IL-1B^+^ macrophages, respectively. Their crosstalk subsequently shaped the tumor microenvironment of HCC, possibly through the CD74-MIF axis. Importantly, patients with high CD74 expression presented higher immune scores and achieved good outcomes after receiving immunotherapy.

**Conclusion:**

High CD74 expression is associated with the abundance of a variety of immune cell types, mediating interactions among tumor and immune cells and shaping the malignant behavior of HCC. In summary, CD74 may be a hallmark for determining the prognosis and immune cell infiltration levels of HCC patients.

**Supplementary Information:**

The online version contains supplementary material available at 10.1186/s10020-024-00884-x.

## Background

Hepatocellular carcinoma (HCC) remains a global challenge for human health (Murai et al. 2023). HCC is a highly aggressive malignancy with a poor prognosis and is the fifth leading cause of cancer-related death worldwide (Siegel et al. 2023). Tumors are composed of a complex tumor microenvironment (TME) that is composed of tumor-infiltrating immune cells, stromal cells, chemokines, and the extracellular matrix (Balkwill et al. 2012). The interactions between immune cells and tumor cells in the TME shape cancer development and influence treatment responsiveness (Binnewies et al. 2018; Shen and Kang 2018). Immune landscape heterogeneity is becoming a key hallmark for determining the progression of HCC (Peneau et al. 2022). Thus, the need to elucidate the underlying mechanism of immune landscape heterogeneity, especially the immunomodulation of the TME in HCC, is urgent.

Based on single-cell omics and spatial transcriptomics techniques, the biology of the TME in HCC has been investigated in detail. Many cell-to-cell crosstalk networks have been reported to drive tumor malignancy (Lei et al. 2021). Cancer-associated fibroblasts are the most common stromal cells, and extensive crosstalk between these cells and immune cells plays a critical role in tumor development (Mao et al. 2021). The interaction between CD36-positive metastasis-associated macrophages and tumor cells controls liver metastasis (Yang et al. 2022). The potential crosstalk between dendritic cells (DCs) and natural killer (NK) cells also affects the progression of HCC (Wang et al. 2022). Therefore, the complex regulatory networks among tumor cells with multiple immune cells are involved in the development of HCC. C-type lectin domain family 9 A (CLEC9A)-cDC1^+^ DCs can regulate tumor immune surveillance and establish an important link between metabolism and immunity, serving as a therapeutic target for skin cancer (Zeng et al. 2023). Hepatic progenitor cells (HPCs) are essential for primary liver tumorigenesis because they affect chronic liver inflammation (Meng et al. 2021). Targeting IL-1B macrophages contribute to the treatment of renal cell carcinoma (RCC) (Li et al. 2022a, b). However, the correlation among these cell types and their interactions with tumor cells in the TME of HCC remain largely unknown.

CD74, a constant chain of major histocompatibility complex II (MHC-II), is mainly responsible for the transport of MHC-II molecules in antigen-presenting DCs and participates in immune regulation (Borghese and Clanchy 2011a, b; Cheng et al. 2015). Studies have shown that CD74 is abnormally expressed in many tumors and is closely related to tumor immune regulation. CD74 is highly expressed in lymphatic cancer and can be used as an important target for immunotherapy in lymphoid/plasmacytoid malignancies (Zhao et al. 2019). CD74 is highly expressed in basal-like breast cancer cell types and is significantly correlated with the number of tumor-infiltrating monocytes, indicating that CD74 is closely related to intratumoral immune regulation (Wang et al. 2017). In metastatic melanoma, blocking macrophage migratory inhibitory factor (MIF)/CD74 signaling in macrophages can reactivate the T-cell immune response and achieve antitumor effects (Figueiredo et al. 2018a, b). Activation of CD74 can block microglial M1 polarization, thus promoting the occurrence and development of glioma (Ghoochani et al. 2016). CD74-positive macrophages are associated with the infiltration of CD8^+^ cytotoxic T lymphocytes (CTLs) in HCC (Xiao et al. 2022). In addition to its effect on immune cells, the inhibition or knockout of CD74 can significantly inhibit the proliferation and invasion of tumor cells in many solid tumors (Meyer-Siegler et al. 2006; Ssadh et al. 2019; Gai et al. 2018). Thus, CD74 can not only directly regulate immune cell activation but also influence tumor progression by regulating tumor cell function. However, how CD74 bridges the crosstalk among tumor cells and immune cells and its function in clinical therapeutics for HCC remain unknown.

In this study, we observed that a high level of CD74 was associated with favorable clinical outcomes in patients with HCC. CD74 mainly influences the progression of HCC by regulating immune response pathways. Additionally, we used single-cell RNA sequencing analysis to delineate the immune landscape and tumor heterogeneity in a cohort of HCC patients with high CD74 expression and low CD74 expression. Our study revealed a potential link between tumor immunity and the expression of CD74 and revealed its important prognostic value for HCC patients receiving immunotherapy.

## Methods and materials

### Data sources

In this research, eleven independent public datasets, namely, the Encyclopedia of RNA Interactomes (ENCORI) database, Tumor Immune Estimation Resource (TIMER), Human Protein Atlas (HPA), Kaplan-Meier (KM), tumor-immune system interactions database (TISIDB), Gene Expression Profiling Interactive Analysis 2 (GEPIA2), LinkedOmics, Comprehensive Analysis on Multi-Omics of Immunotherapy in Pan-cancer (CAMOIP), Tissue-specific Gene Expression and Regulation (TIGER), single-cell transcriptomes of tumor immune microenvironment (SCTIME) and Tumor Immune Single-cell Hub 2 (TISCH2), were searched to analyze the role of CD74 in the TME of HCC.

### Differential expression analysis

The differential transcription and expression of CD74 in HCC samples and normal samples was analyzed using the ENCORI and TIMER databases. Immunohistochemical results were obtained from the HPA database, and fresh HCC samples were stained with an anti-CD74 antibody. Differentially expressed genes (DEGs) in CD74^high^ and CD74^low^ HCC patients were analyzed using the CAMOIP database. CD74 expression data in different cell populations were obtained from the TIGER database. The differential expression of CD74 in HCC patients who underwent immunotherapy was analyzed using a receiver operating characteristic (ROC) curve. Gene expression associations were assessed via the GEPIA2 database.

### Survival analysis

KM Plotter was used to analyze the overall survival (OS) of CD74^high^ and CD74^low^ HCC patients who consumed alcohol or had hepatitis virus infection. The OS of HCC patients who underwent immunotherapy was obtained from ROC curves.

### Immunohistochemical (IHC) staining

The HCC tissue microarray chips were purchased from OUTDO Biotechnology Co., Ltd. (Shanghai, China). All experiments were approved by the Ethics Committee of Xi’an Medical University. First, the sections were dewaxed and rehydrated and subsequently subjected to antigen retrieval using a high-pressure method. H_2_O_2_ (3%) was used to block endogenous peroxidase activity. After blocking with 10% bovine serum albumin (BSA) for 1 h, the sections were incubated with primary antibodies against CD74 (1:50, Abcam, ab9514, UK) overnight at 4 °C. The next day, the slices were incubated with an HRP-conjugated secondary antibody at room temperature for 1 h. The nuclei were stained with DAPI and visualized after staining with DAB (ZL1-9081, ZSGB-BIO, China), and images were acquired with an inverted biological microscope (IX51, Olympus, Japan). Comprehensive analyses, including staining intensity and the number of positive cells, were conducted using Image-Pro Plus 6.0 software.

### LinkedOmics analysis

The LinkedOmics database (http://www.linkedomics.org) was used to analyze multiomics data and clinical data from HCC patients. The LinkFinder module of LinkedOmics showed DEGs in HCC that were correlated with CD74 in the Liver Hepatocellular Carcinoma (LIHC) cohort (*n* = 371, ID-146160). All data were investigated by calculating Pearson’s correlation coefficient and are presented as heatmaps. Pathways and networks composed of DEGs can be found under the LinkInterpreter module. After signing and ranking, gene set enrichment analysis (GSEA) was performed to elucidate GO (CC: cellular component, BP: biological process, and MF: molecular function) and KEGG pathways. The rank criterion from the LinkFinder results was an FDR < 0.05, and 500 simulations were selected.

### Immune cell infiltration analysis

Correlations between the copy number variation (CNV) of CD74 and the abundances of six types of tumor-infiltrating immune cells (TIICs) (B cells, CD4 + T cells, CD8 + T cells, neutrophils, macrophages, and dendritic cells) were explored using the TIMER database (https://cistrome.shinyapps.io/timer/). Then, a correlation module was used to evaluate the correlation between CD74 expression and immune cell abundance. The distributions of 22 subtypes of immune cells in the low and high CD74 expression groups were analyzed using the CAMOIP database. The associations between tumor mutational burden (TMB) and CD74 expression and between neoantigen expression and CD74 expression were explored via the CAMOIP database. The immune cell score was determined by the CAMOIP database.

### Single-cell analysis

The differential expression of CD74 in immune cell types and the differentiation of myeloid cells were explored using the TIGER database. The interaction network among immune cells and malignant cells was determined by using the SCTIME database. The associations between CD74 expression in HPCs or malignant cells and the abundances of DCs and macrophages were also analyzed via the SCTIME database.

### Cell-to‐cell communication analysis

CellChat (v1.1.3) was used to infer cell-to-cell interactions between IL-1B^+^ macrophages and malignant cells and between CLEC9A-cDC1^+^ DCs and HPCs via the SCTIME database. The expression distribution of CD74 in different cell populations was determined with the TISCH2 database. The enriched signaling pathways in CD74^high^ and CD74^low^ HCC patients were identified by ssGSEA using the CAMOIP database.

### Statistical analysis

All data were obtained from the Bioinformatics Online Database. The significance of the differential expression of CD74 in LIHC tissues was determined by Student’s t test. The log-rank test was used to determine the significance of differences in survival time. The Kruskal test was applied to investigate the associations between CD74 expression and cell types. Pearson’s correlation coefficient was used to determine how closely related the genes were. A threshold of *p* < 0.05 indicated the significance of all analyses.

## Results

### A high level of CD74 indicates a good prognosis for patients with LIHC

**Fig. 1:**
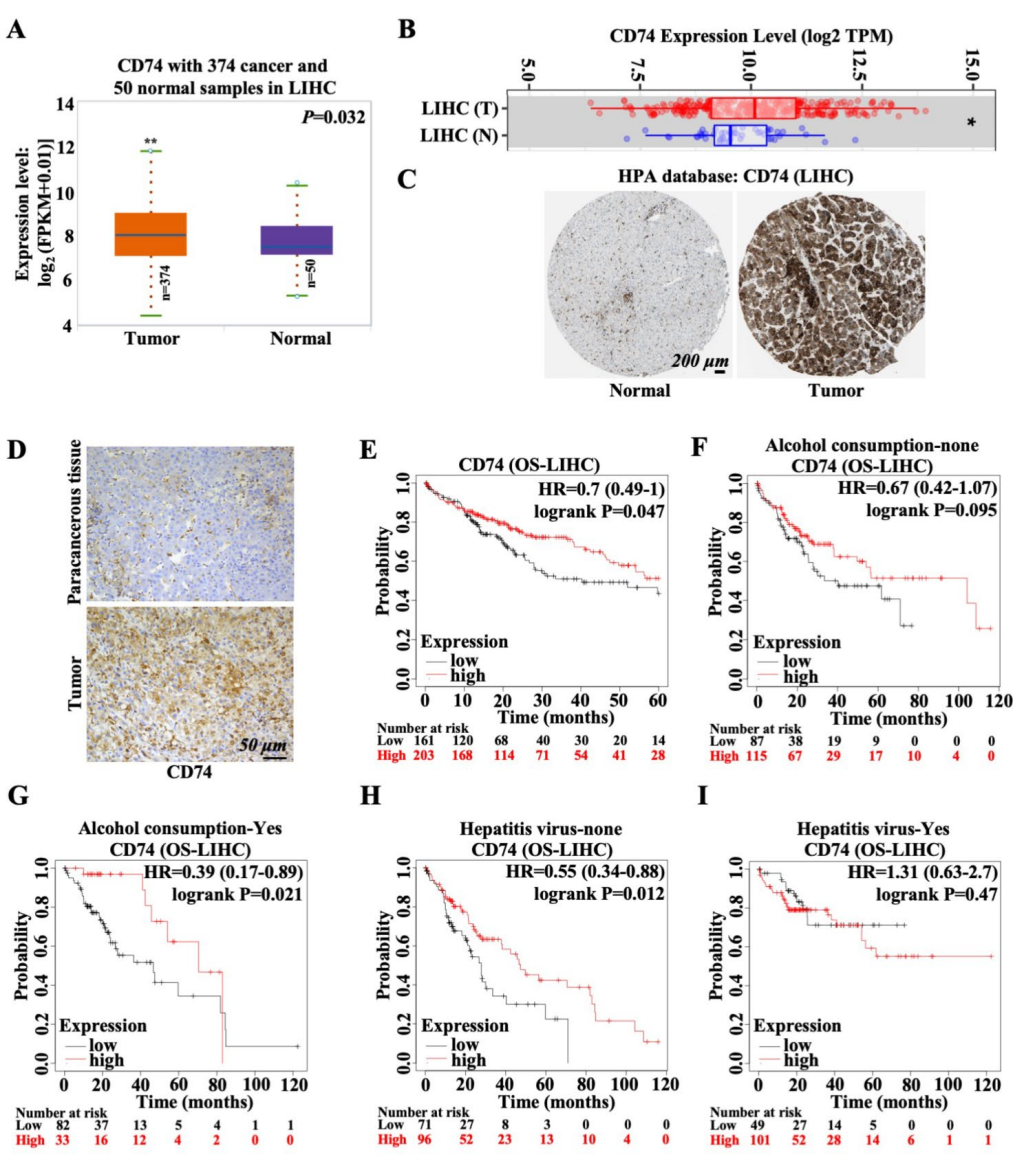
Differential expression and clinical prognosis analysis of CD74 expression in LIHC. **(A)** Box plot from the Encyclopedia of RNA Interactomes (ENCORI) database showing CD74 mRNA levels in cancer (*n* = 374) and normal (*n* = 50) liver hepatocellular carcinoma (LIHC) samples. **(B)** Box plot from the Tumor Immune Estimation Resource (TIMER) database showing CD74 mRNA levels in LIHC tumor and normal tissue samples. **(C)** CD74 protein levels in normal liver and LIHC tissues were visualized by immunohistochemical (IHC) staining in the Human Protein Atlas (HPA) database. Scale bar: 200 μm. **(D)** CD74 protein levels in paracancerous tissues and tumor tissues from fresh HCC tissue samples were visualized by IHC. Scale bar: 50 μm. **(E)** OS curve according to differential CD74 expression in LIHC patients. **(F-G)** OS curves stratified by differential CD74 expression in LIHC patients without alcohol consumption (F) and in LIHC patients with alcohol consumption (G). **(H-I)** OS curves according to differential CD74 expression in LIHC patients without hepatitis virus infection (H) and in LIHC patients with hepatitis virus infection (I). *, *p* < 0.05; **, *p* < 0.01

The CD74 transcription level was first analyzed using the ENCORI and TIMER databases. The data from both databases showed that CD74 was more highly expressed in LIHC tissues than in normal tissues (Fig. 1A and B). Consistent with these findings, immunohistochemical data from the HPA database and immunohistochemical staining results showed that the CD74 protein level in LIHC tissues was notably greater than that in normal tissue or para-cancerous tissues (Fig. 1C and D). However, CD74 expression did not change among LIHC patients with different disease stages (stage I, II, III and IV) (Figure S1A). Subsequently, the association between survival rates and CD74 expression in different LIHC patients was evaluated using Kaplan-Meier survival curves. As shown in Fig. 1E, the OS of LIHC patients in the low CD74 expression group (CD74^low^) was significantly shorter than that of patients in the high CD74 expression group (CD74^high^) (Fig. 1E), which indicated that CD74 might be a promising biomarker for predicting survival in LIHC patients. In particular, CD74^high^ LIHC patients who consumed alcohol or CD74^high^ LIHC patients without hepatitis virus infection had better prognoses than did CD74^low^ patients (Fig. 1G and H). However, these differences between the CD74^high^ group and the CD74^low^ group were not observed in LIHC patients who did not consume alcohol or in LIHC patients with hepatitis virus infection (Fig. 1F and I). Additionally, we compared the differential expression of CD74 between alcohol vs. nonalcohol populations and between hepatitis-positive and hepatitis-negative populations in patients with HCC. High levels of CD74 were detected in alcohol-consuming and hepatitis virus-positive HCC samples compared to non-alcohol-consuming and hepatitis virus-negative HCC samples (Figure S1B-S1C). Although the differences were significant between them, the specific expression levels were slight (14.84 vs. 14.54 and 14.86 vs. 14.62). Thus, the difference in predicting overall survival rate has little to do with CD74 levels in alcohol vs. nonalcohol or hepatitis-positive vs. hepatitis-negative populations. Collectively, these data indicate that the CD74 expression level may predict the prognosis of patients with LIHC, particularly in alcohol drinkers or hepatitis virus-negative populations.

### CD74 and its coexpressed genes are enriched in the immune response in LIHC tissues

**Fig. 2:**
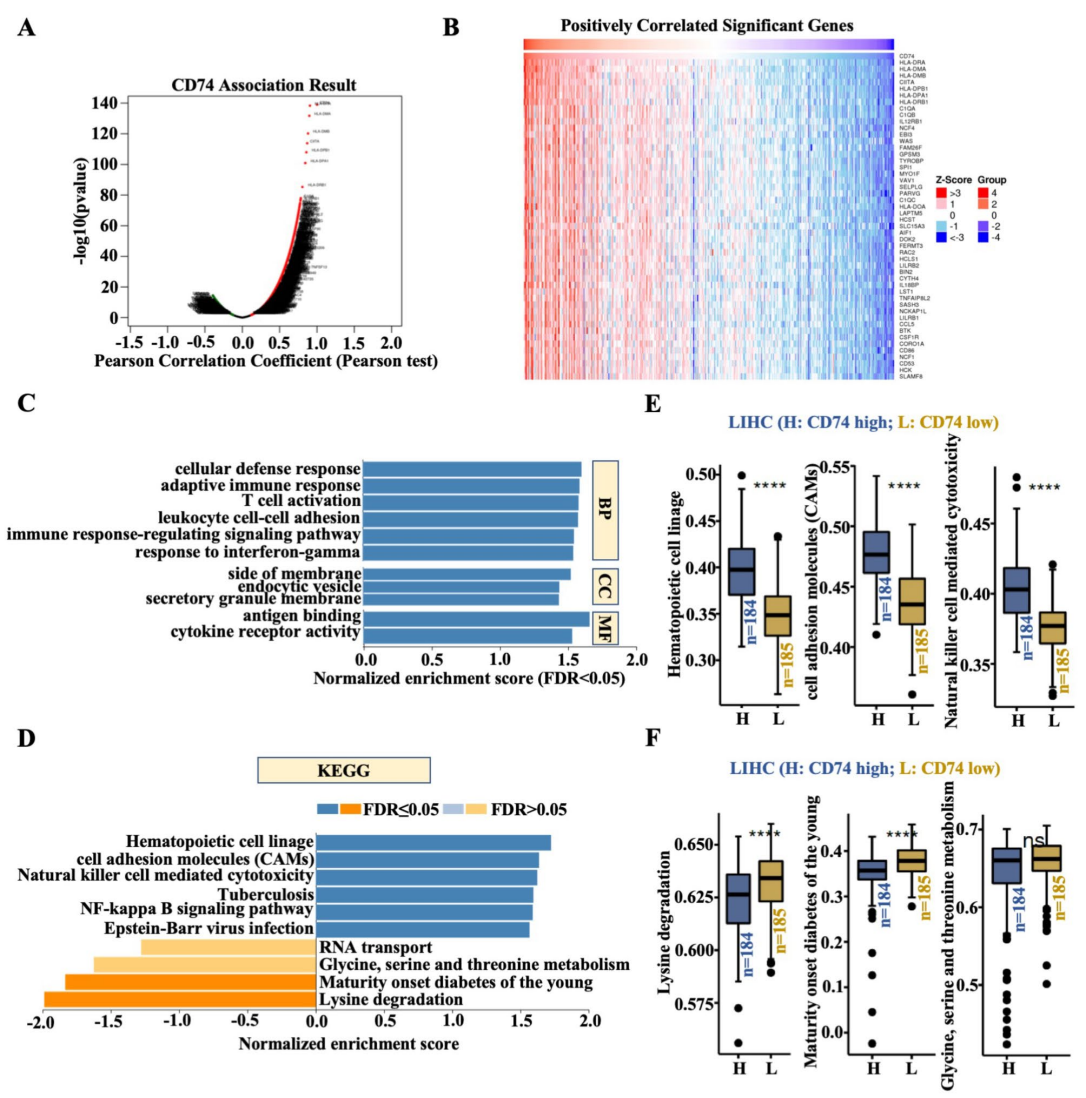
GO annotation/KEGG pathway analyses of genes coexpressed with CD74 in LIHC. **(A)** Volcano plot showing genes positively and negatively associated with CD74 in LIHC. **(B)** Heatmaps showing genes associated with CD74 in LIHC (top 50). Red indicates a positive gene correlation, and green indicates a negative gene correlation. **(C)** The GO annotations of genes coexpressed with CD74 in liver tissue were analyzed by gene set enrichment analysis (GSEA). BP, Biological processes; CC, Cellular components; MF, Molecular functions. **(D)** KEGG pathway analysis of genes coexpressed with CD74 in LIHC as analyzed by GSEA. The FDR from GSEA was 0. **(E-F)** The positively (E) and negatively (F) enriched pathways with respect to CD74 differential expression in LIHC as confirmed via ssGSEA by Comprehensive Analysis on Multi-Omics of Immunotherapy in Pan-cancer (CAMOIP) database. ****, *p* < 0.0001

CD74 coexpressed genes (genes identified in both LIHC tissues and GO and KEGG pathway analyses) were analyzed using LinkedOmics. Combined with the LinkedOmics database, CD74 coexpressed genes, as shown in the volcano plot, were screened, and the top 50 genes that were positively and negatively related to CD74 are presented as heatmaps (Fig. 2A and B and Figure S2). The top-ranked positively related genes were principally members of the human leukocyte antigen (HLA) family, such as HLA-DRA (DR alpha chain), HLA-DMA (DM alpha chain), HLA-DMB (DM beta chain), HLA-DPB1 (DP beta chain 1), HLA-DPA1 (DP alpha chain 1) and HLA-DRB1 (DR beta chain 1) (Fig. 2B). GO analysis via GSEA revealed that the genes coexpressed with CD74 were involved mainly in the cellular defense response, the adaptive immune response, T-cell activation, leukocyte cell-cell adhesion, the immune response-regulating signaling pathway, and the response to interferon-gamma (BP analysis). The data suggested that CD74 coordinated with other genes and mainly participated in immune response-related biological processes. The coexpressed genes were principally located on the side of the membrane, within endocytic vesicles, and on the secretory granule membrane (CC analysis). Molecular function (MF) analysis revealed that antigen binding and cytokine receptor activity were significantly related to CD74-related genes (Fig. 2C). KEGG pathway analysis revealed that the enrichment pathways of the genes coexpressed with CD74 were involved in the hematopoietic cell lineage, cell adhesion molecules (CAMs), natural killer cell-mediated cytotoxicity, tuberculosis, the NF-kappa B signaling pathway and Epstein–Barr virus infection. The pathways negatively associated with the genes coexpressed with CD74 were the lysine degradation pathway and the maturity-onset diabetes of the young pathway (Fig. 2D). Additionally, single sample gene set enrichment analysis (ssGSEA) was subsequently performed to verify the results of the KEGG analysis. CD74^high^ LIHC patients (*n* = 184) exhibited more hematopoietic cell lineage, cell adhesion molecule (CAM) and natural killer cell-mediated cytotoxicity pathway activity than did CD74^low^ LIHC patients (*n* = 185) (Fig. 2E), which indicated that CD74 and its coexpressed genes were involved in the process of natural killer cell-mediated innate immunity. In contrast, compared with CD74^low^ LIHC patients, CD74^high^ LIHC patients exhibited decreased lysine degradation and maturity-onset diabetes of the young pathway activity (Fig. 2F). Consistent with these findings, there were no changes in the glycine, serine and threonine metabolism pathway between the CD74^high^ and CD74^low^ LIHC patients (Fig. 2D and F). Taken together, these findings indicate that CD74 is an important immune regulator in LIHC.

### CD74 is positively associated with CD8^+^ T-cell infiltration and negatively associated with M2 macrophage infiltration

**Fig. 3:**
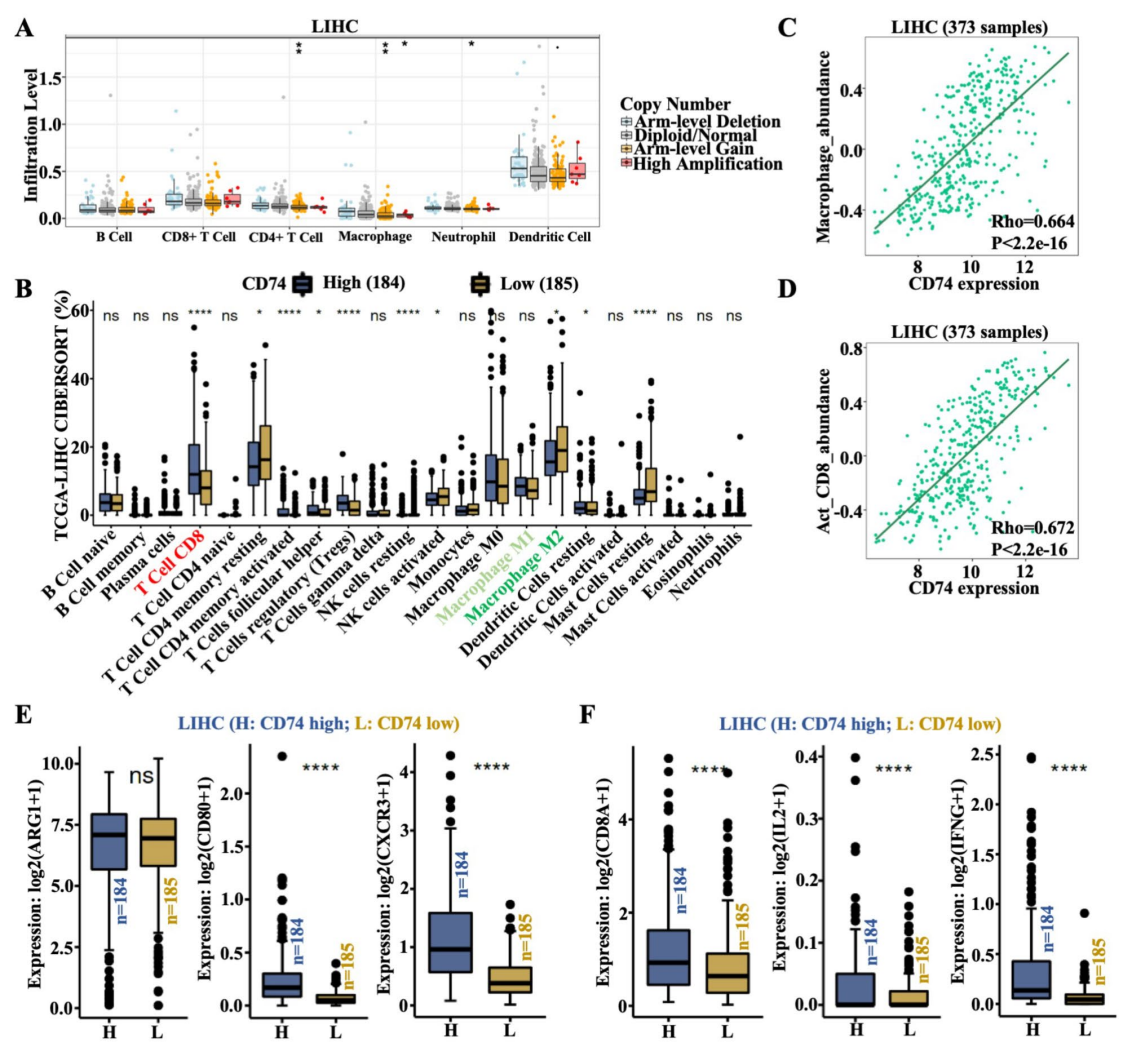
Relationships between CD74 expression and immune infiltration levels in LIHC. **(A)** Association between CD74 copy number variation (CNV) and the infiltration levels of B cells, CD8^+^ T cells, CD4^+^ T cells, macrophages, neutrophils and dendritic cells (DCs) in liver tumor tissues. **(B)** The distribution of 22 subtypes of immune cells in the low and high CD74 expression groups. **(C-D)** Association between CD74 expression and macrophage abundance (C) and between CD74 expression and CD8^+^ T-cell abundance (D) in LIHC according to the TIMER database. **(E)** Gene expression levels of the M2 macrophage marker ARG1 and the M1 macrophage markers CD80 and CXCR3 in liver tumor tissues with high CD74 (*n* = 184) or low CD74 (*n* = 185) expression, as determined by the CAMOIP database. **(F)** Gene expression levels of the activated CD8^+^ T-cell markers CD8A, IL-2 and IFNG in liver tumor tissues with high CD74 (*n* = 184) or low CD74 (*n* = 185) expression, as determined by the CAMOIP database. *, *p* < 0.05; **, *p* < 0.01; ****, *p* < 0.0001

Subsequently, the TIMER database was used to determine whether CD74 expression in liver cancer was related to the level of immune cell infiltration. The data indicated that the CNV of CD74 was notably related to the infiltration levels of CD4^+^ T cells, macrophages and neutrophils, and high amplification of CD74 was only related to the infiltration level of macrophages (Fig. 3A). Combined with TCGA-LIHC CIBERSORT data, the differences in 22 types of TIICs were compared between CD74^high^ and CD74^low^ LIHC patients. The data showed that CD74^high^ patients possessed more CD8^+^ T cells, activated CD4^+^ memory T cells, follicular helper T cells, regulatory T cells (Tregs), and resting DCs and fewer resting CD4^+^ memory T cells, resting natural killer (NK) cells, activated NK cells, M2 macrophages, and resting mast cell infiltrates (Fig. 3B). Unlike in the TIMER database, in the TISIDB database, both CD4^+^ memory T-cell and CD4^+^ T-cell abundances were positively associated with CD74 expression in LIHC (Figure S3A-S3B). Given the different immune regulatory effects and the significance of the changes, three cell types, CD8^+^ T cells, Tregs and macrophages, were selected to further evaluate the role of CD74 in the TME. Based on the TISIDB, significant positive associations between CD74 expression and macrophage, CD8^+^ T-cell and Treg abundances were observed (Fig. 3C and D and Figure S3C). Next, the CAMOIP database was used to assess the immune infiltration of the three cell types. Although the expression of the M2 macrophage marker gene ARG1 was not significantly different between CD74^high^ and CD74^low^ LIHC patients, there was a marked increase in the expression of the M1 macrophage marker genes CD80 and CXCR3 in CD74^high^ LIHC patients compared to that in CD74^low^ LIHC patients, which indirectly reflected the decrease in the M2 macrophage population in the CD74^high^ population (Fig. 3E). Consistent with the above results, CD8^+^ T-cell marker genes, including CD8A, IL-2, and interferon gamma (IFNG), and Treg marker genes, including Forkhead box protein P3 (FOXP3), cytotoxic T lymphocyte-associated protein 4 (CTLA4), and interleukin-2 receptor alpha chain (IL-2RA), were all obviously enhanced in the CD74^high^ population (Fig. 3F and Figure SD). Due to the opposite effects of CD8^+^ T cells and Tregs in the TME, the effect of CD74 on macrophage function was subsequently chosen for further in-depth analysis. In view of the decrease in M2 macrophages and moderate increase in M1 macrophages in the CD74^high^ population, an immune-activated TME may develop in HCC patients with high CD74 expression through the blockade of M2 polarization.

### CD74 determines the differentiation process of myeloid cells

**Fig. 4:**
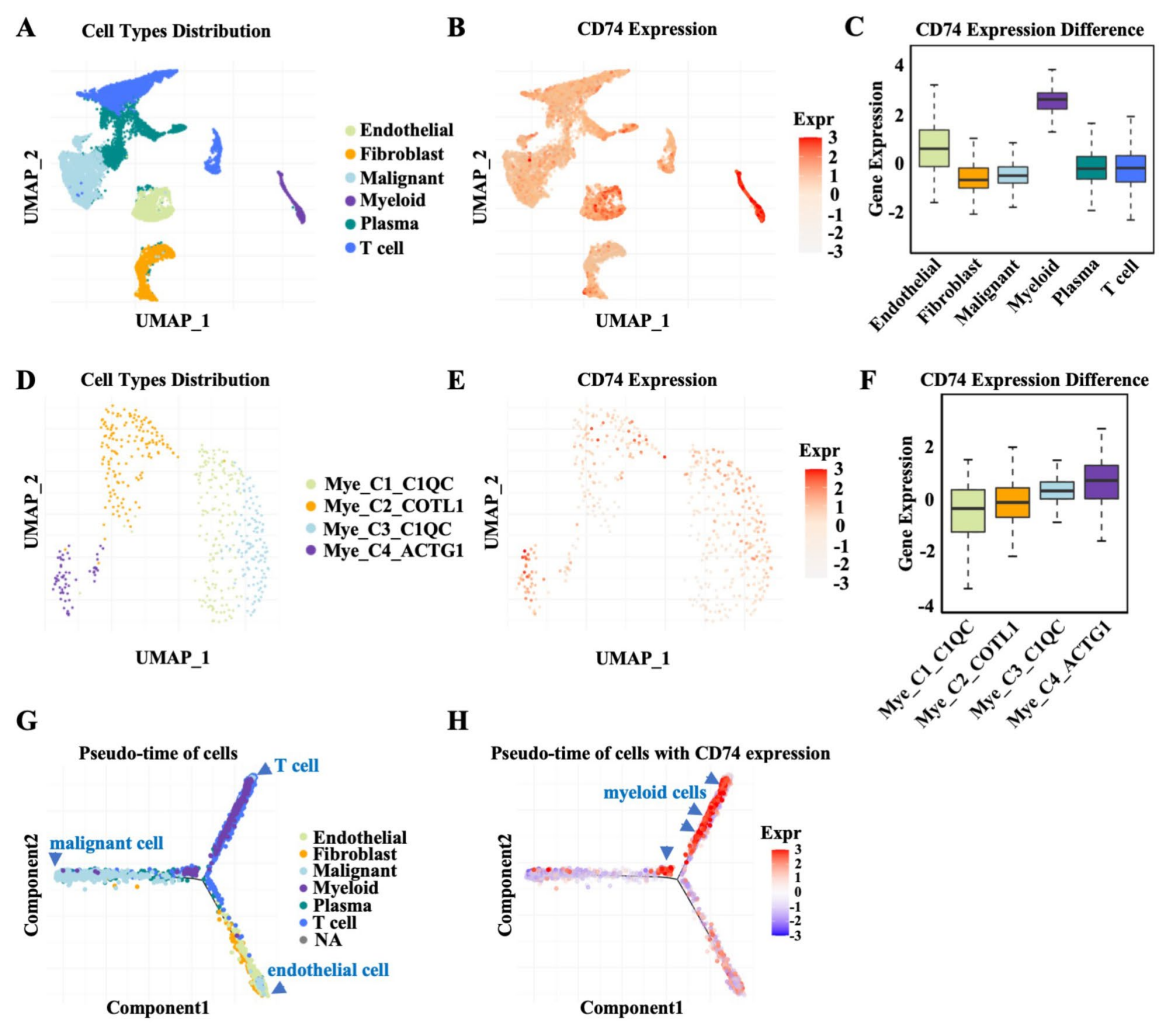
Delineation of the cell type distribution in LIHC using scRNA-seq datasets from open-source databases. **(A)** UMAP plot showing the main cell types, including endothelial cells, fibroblasts, malignant cells, myeloid cells, plasma cells and T cells, in LIHC tissues. **(B)** UMAP plot showing the distribution of CD74 expression in endothelial cells, fibroblasts, malignant cells, myeloid cells, plasma cells and T cells. **(C)** Histogram visually displaying differences in CD74 expression in endothelial cells, fibroblasts, malignant cells, myeloid cells, plasma cells and T cells. **(D)** UMAP plot showing the landscape of myeloid cells, including C1_C1QC^+^ myeloid cells, C2_COTL1^+^ myeloid cells, C3_C1QC^+^ myeloid cells and C4_ACTG1^+^ myeloid cells. **(E)** UMAP plot showing the CD74 expression distribution in C1_C1QC^+^ myeloid cells, C2_COTL1^+^ myeloid cells, C3_C1QC^+^ myeloid cells and C4_ACTG1^+^ myeloid cells. **(F)** Histogram visually displaying differences in CD74 expression among C1_C1QC^+^ myeloid cells, C2_COTL1^+^ myeloid cells, C3_C1QC^+^ myeloid cells and C4_ACTG1^+^ myeloid cells. **(G)** Pseudotime analysis of the trajectory of endothelial cells, fibroblasts, malignant cells, myeloid cells, plasma cells and T cells in LIHC tissues. Each dot in the tree structure represents one cell, which is color coded according to its cell type. **(H)** Association between CD74 expression and the pseudotime distribution of endothelial cells, fibroblasts, malignant cells, myeloid cells, plasma cells and T cells in LIHC tissues

To more deeply explore the role of CD74 in the innate immune system of macrophages, the TIGER database was used to determine the association between CD74 expression and the heterogeneous single-cell landscapes of LIHC. The cell populations were divided into six main cell types: endothelial cells, fibroblasts, malignant cells, myeloid cells, plasma cells and T cells (Fig. 4A). CD74 was expressed in all cell types, particularly in endothelial cells and myeloid cells, with the highest level observed in myeloid cells (Fig. 4B and C). Myeloid cells were selected for further analysis because they are a source of macrophages. The myeloid cells were divided into four population groups according to the expression of C1_C1QC (component 1, q subcomponent, C chain), C2_COTL1 (coactosin-like 1), C3_C1QC and C4_ACTG1 (actin gamma 1) (Fig. 4D). The data indicated that the highest expression of CD74 mainly appeared in the C4_ACTG1 myeloid cell population, which was associated with multilevel immune cell infiltration, compared to the other three cell population groups, which further proved the regulatory role of CD74 in immune infiltration (Fig. 4E and F). Importantly, pseudotime analysis was performed to assess cell development in LIHC. The two-dimensional tree structure began with malignant cells and ended with endothelial cells or T cells, indicating two development paths in LIHC (P1: malignant cells to endothelial cells; P2: malignant cells to T cells) (Fig. 4G). CD74 expression was mainly enriched in myeloid cells, indicating that CD74 is involved in the differentiation of myeloid cells (Fig. 4H). Our data further confirmed the critical role of CD74 in the differentiation of myeloid cells.

### CD74 expression in malignant or HPC-like cells affects the population of myeloid-derived cells

**Fig. 5:**
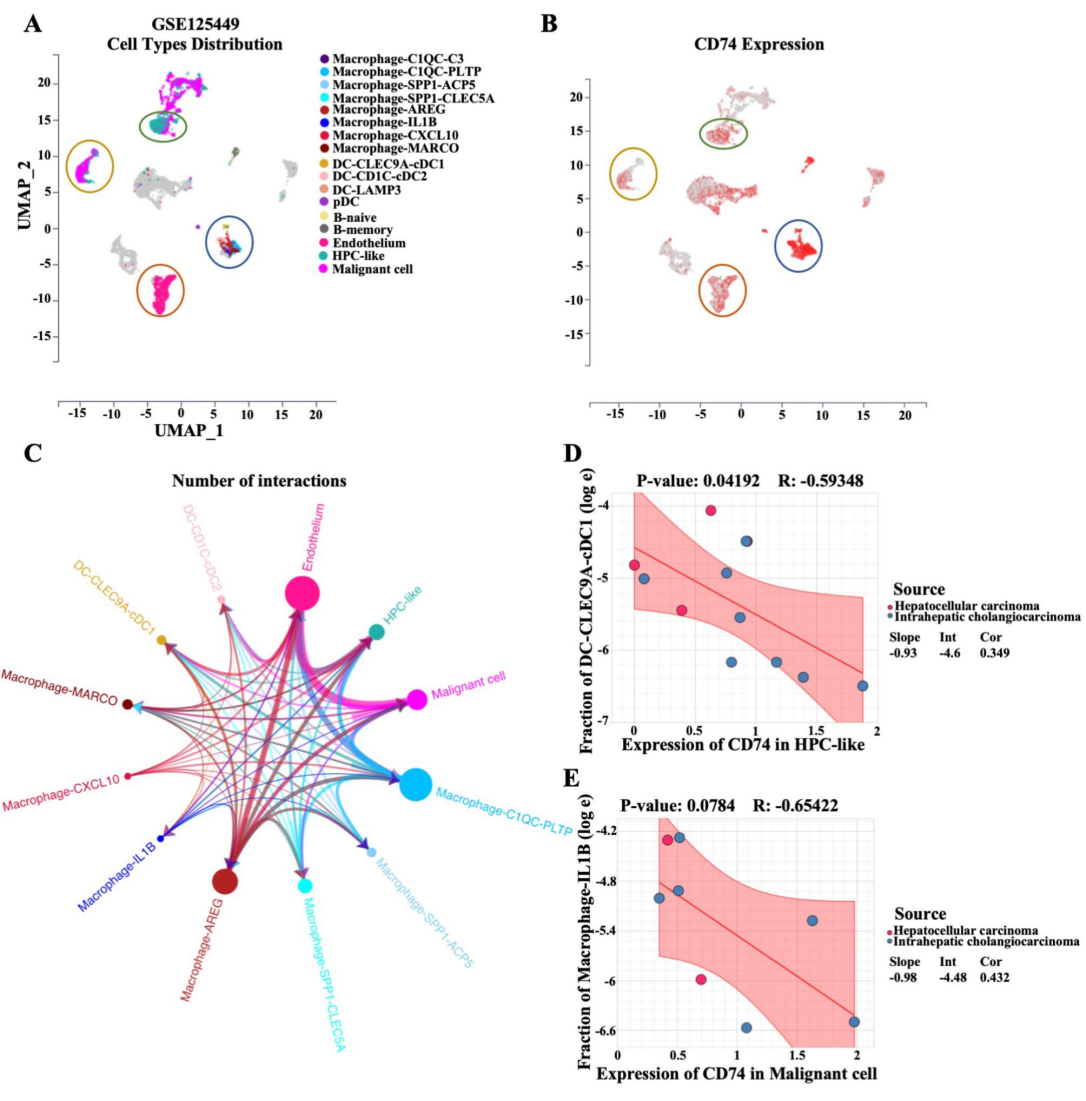
Cell-to‐cell communication analysis in LIHC patients with differential CD74 expression. **(A)** UMAP plot showing the cell type distribution combined with single-cell data retrieved from the Gene Expression Omnibus (GSE125449). **(B)** Single-cell transcriptomic data of CD74 in different cell types were retrieved from GSE125449. **(C)** Circle plots showing an overview of cell–cell interactions among endothelial cells, DCs, malignant cells and macrophages based on single-cell RNA-seq data. **(D)** Correlation analysis of CD74 expression in hepatic progenitor cells (HPCs) and the cell composition of CLEC9A-cDC1^+^ DCs. **(E)** Correlation analysis of CD74 expression in malignant cells and the cell composition of IL-1B^+^ macrophages

A previously published single-cell dataset (GSE125449) was used to determine the effect of CD74 on myeloid-derived cells. The cell populations were divided into fifty main cell types (Figure S4A). The data demonstrated that CD74 was mainly expressed in macrophages, DCs, and B cells that were all derived from myeloid cells and was also expressed in endothelial cells, HPC-like cells and malignant cells (Fig. 5A and B). The circle plots showed that HPC-like cells and malignant cells had the greatest number of interactions with other stromal cells and macrophages in LIHC tumors, indicating their active biological properties (Fig. 5C). Expression and cell composition correlation analysis revealed that patients with high CD74 expression in HPC-like cells exhibited a smaller fraction of CLEC9A-cDC1^+^ DCs than did those with low CD74 expression in HPC-like cells, which suggested that the priming effect of CLEC9A-cDC1^+^ DCs on anticancer CD8^+^ T cells was restrained by high CD74 expression in HPC-like cells (Fig. 5D). Additionally, the fraction of IL-1B^+^ macrophages decreased with increasing CD74 expression in malignant cells, which indicated a decreased proinflammatory effect of IL-1B^+^ macrophages (Fig. 5E). However, there were no significant changes in the fraction of HPC-like cells or malignant cells among CD74^high^ CLEC9A-cDC1^+^ DCs or CD74^high^ IL-1B^+^ macrophages, respectively (Figure S4B-S4C). These findings indicate that CD74 in HPC-like or malignant cells might affect the abundance of myeloid-derived immune cells (especially CLEC9A-cDC1^+^ DCs and IL-1B^+^ macrophages) and cell–cell interactions, resulting in changes in immune activity in the TME.

### The CD74-MIF signaling axis mediates cell–cell interactions among HPC-like cells, DCs, malignant cells and macrophages

**Fig. 6:**
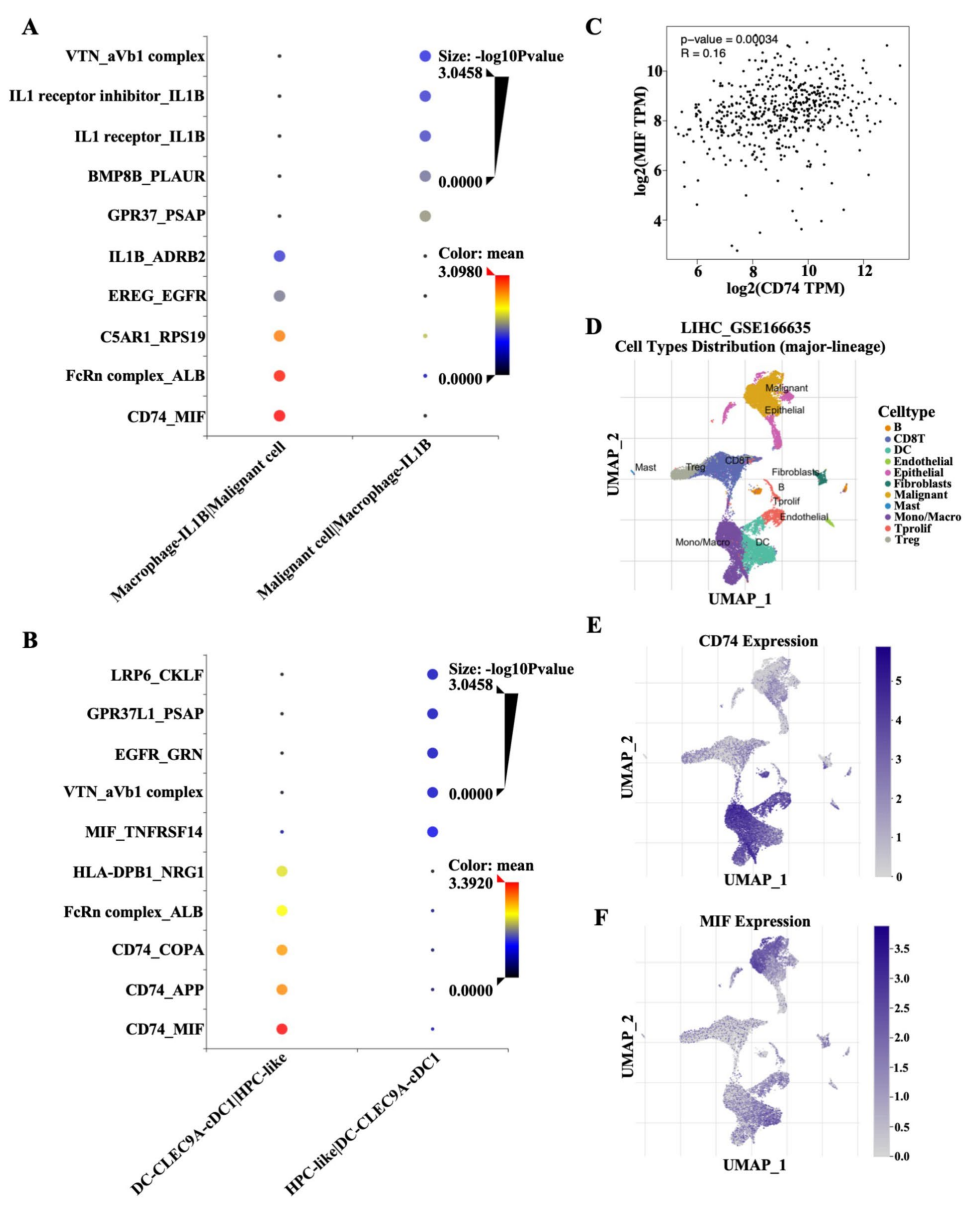
Ligand-receptor (LR) network analysis of the crosstalk among DCs, HPC-like cells, malignant cells and macrophages. **(A)** Dot plot showing the LR analysis between IL-1B^+^ macrophages and malignant cells. **(B)** Dot plot showing the LR analysis between CLEC9A-cDC1^+^ DCs and HPC-like cells. **(C)** Association analysis between CD74 expression and MIF expression in LIHC. **(D)** UMAP plot showing the cell type distribution (major lineage) combined with single-cell data retrieved from GSE166635. **(E)** Single-cell transcriptomic data of CD74 in different cell types were retrieved from GSE166635. **(F)** Single-cell transcriptomic data of MIF in different cell types were retrieved from GSE166635.

MIF signals are reportedly associated with immunological escape from the TME. In this study, IL-1B^+^ macrophages highly expressed CD74 or the neonatal Fc receptor (FcRn) complex to interact with MIF and ALB in malignant cells, respectively (Fig. 6A). In addition, CLEC9A-cDC1^+^ DCs highly expressed CD74 to interact with MIF, amyloid precursor protein (APP) and coatomer protein complex subunit alpha (COPA) of HPC-like cells (Fig. 6B). Thus, CD74 in combination with MIF serves a key regulatory role in the interactions among HPC-like cells, cancer cells and innate immune cells. According to the GEPIA2 database, a significant positive association between CD74 and MIF was observed in LIHC (Fig. 6C). Our data also indicated that CD74 was mainly enriched in macrophages/monocytes and DCs and that MIF was mainly expressed in malignant cell populations according to a previously published single-cell dataset (GSE166635) (Fig. 6D and F). These results indicate the involvement of CD74-MIF in cell–cell interactions between HPC-like cells and CLEC9A-cDC1^+^ DCs or between malignant cells and IL-1B^+^ macrophages.

### High levels of CD74 facilitate immunotherapy in LIHC

**Fig. 7:**
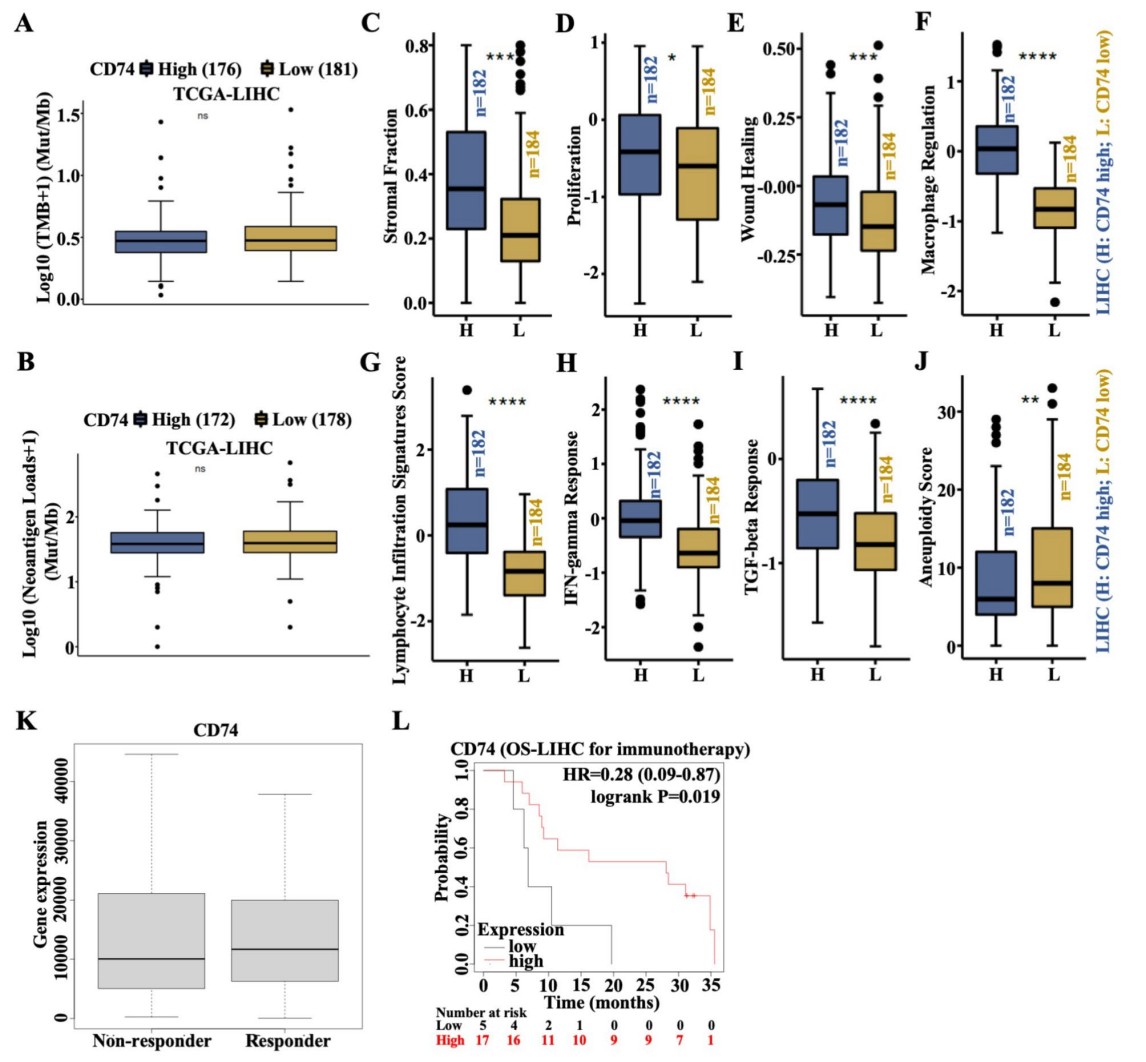
Immune scores of LIHC patients with differential CD74 expression. (**A**) Analysis of differences in the tumor mutational burden (TMB) between CD74-high and CD74-low LIHC patients. **(B)** Differential analysis of neoantigens in patients with CD74-high and CD74-low LIHC. **(C-J)** Immune scores, including the stromal fraction (**C**), proliferation (**D**), wound healing (**E**), macrophage regulation (**F**), lymphocyte infiltration signature score (**G**), IFN-gamma response (**H**), TGF-beta response (**I**), and aneuploidy score (**J**), in patients with high CD74 expression and low CD74 expression in LIHC. (**K**) Differences in CD74 expression between nonresponder and responder LIHC patients after receiving immunotherapy. (**L**) OS curve according to differential CD74 expression in LIHC patients who received immunotherapy. *, *p* < 0.05; **, *p* < 0.01; ***, *p* < 0.001; ****, *p* < 0.0001

Since CD74 can shape the TME (especially myeloid-derived cells) in LIHC, we subsequently assessed the immune scores of LIHC patients with differential CD74 expression. First, the data demonstrated that there was no significant association between CD74 expression and the TMB in LIHC patients (Fig. 7A). In addition, neoantigen load was not associated with CD74 levels in LIHC (Fig. 7B). However, immune score indices, including the stromal fraction, proliferation, wound healing, macrophage regulation, lymphocyte infiltration signature score, IFN-gamma response, and TGF-beta response, were all notably greater in the CD74^high^ LIHC patients than in the CD74^low^ LIHC patients (Fig. 7C and I). In contrast, immune score indices, such as the aneuploidy score, sharply decreased in CD74^high^ LIHC patients (Fig. 7J). A high immune score is reportedly associated with a good prognosis after receiving immunotherapy. KM analysis revealed that CD74 was more highly expressed in responders than in nonresponders among LIHC patients after immunotherapy (Fig. 7K). Additionally, better prognosis was observed in CD74^high^ LIHC patients than in CD74^low^ LIHC patients after receiving immunotherapy (Fig. 7L). However, MIF expression did not affect the survival rate of patients with LIHC after receiving immunotherapy (Figure S5). Overall, high CD74 expression is associated with greater immune scores in LIHC patients and predicts a good prognosis in LIHC patients receiving immunotherapy.

## Discussion

Although therapeutically targeting the TME is hypothesized to be effective in achieving precision treatment, most therapeutic strategies still fail to suppress tumor progression because of intratumoral heterogeneity (Chen et al. 2020). Thus, there is an urgent need to identify the interactions among tumor-specific cell populations that perform important functions and dissect biological events that occur in the TME. Understanding the TME may facilitate the development of new immune therapy strategies for HCC malignancy.

CD74 is highly expressed in the tumor tissue of diethylnitrosamine/CCl_4_-treated C57BL/6 mice, and CD74 in tumor cells exerts pro-carcinogenic effects by triggering fibrogenesis during HCC development (Wirtz et al. 2021). CD36^+^ CAFs regulate the immune evasion of HCC cells by secreting MIF and upregulating CD74 expression (Yang et al. 2022). In this study, we revealed that high levels of CD74 were present in LIHC patients, which was consistent with previous results in mice. A previous study indicated that stromal CD74^+^ cell enrichment is associated with favorable prognosis in HCC patients (Xiao et al. 2022). Similarly, our data revealed that patients with high CD74 expression presented better prognosis than did those with low CD74 expression. However, whether there is a difference in the ability of high CD74 expression in cancer cells and immune cells to predict patient outcome remains unclear. HCC mainly evolves from diverse chronic liver diseases, and the risk factors include hepatitis B/C virus infection (HBV and HCV), alcohol abuse and other metabolic or genetic abnormalities (Li et al. 2022a, b). Uniquely, better outcomes were observed for patients with high CD74 expression who consumed alcohol and who were not infected with hepatitis virus. The data suggested that risk factors including alcohol abuse and hepatitis virus infection can affect the prognostic value of CD74 in HCC patients. CD74 enhances neuroplasticity in the tumor microenvironment of pancreatic ductal adenocarcinoma via a complex signaling axis (Zhang et al. 2021). As an invariant chain, CD74 participates in several key processes of the immune system, including antigen presentation, B-cell differentiation and inflammatory signaling, in many cancer types (Borghese and Clanchy 2011a, b). Our data revealed several upregulated DEGs in LIHC that were positively correlated with CD74. These DEGs were mainly related to human leukocyte antigen (HLA)-related genes. These DEGs coexpressed with CD74 were mostly enriched in immune response-related biological processes. These findings indicate that the ectopic expression of CD74 may regulate HCC progression by affecting the immune system and cell-to-cell communication in the TME.

CD74 is also highly involved in antigen presentation and the activation of CD4^+^ T cells (Cresswell 1994). Herein, we found that the CNV of CD74 was also correlated with the infiltration of CD4^+^ T cells and macrophages in HCC, which indicated that CD74 regulates immune infiltration. High infiltration of CD74^+^ macrophages was positively associated with the infiltration of CD8^+^ T cells in HCC (Xiao et al. 2022). In glioblastoma, tumor-associated macrophages tended to polarize toward M2 macrophages in the high CD74 group (Wang et al. 2023). Our data indicated that patients with high CD74 expression had more CD8^+^ T cells and M1 macrophages, indicating stronger antitumor activity, which differed from observations in glioblastoma. The enrichment of chemokine (C-C motif) ligand 13-positive myeloid cells mediated immunosuppression through the MIF-CD74 signaling axis (Du et al. 2022). We demonstrated that CD74 was mainly expressed in myeloid cells, especially in C4_ACTG1^+^ myeloid cells, in HCC. ACTG1 was associated with multilevel immune cell infiltration (Zhong et al. 2023). Thus, CD74 may shape the TME by regulating the biological processes of myeloid cells.

CD74 exerts diverse biological functions in physiological and pathological situations, including T-cell and B-cell development, DC motility, macrophage inflammation, and thymic selection (Su et al. 2017). Our findings revealed that CD74 is an important factor in the differentiation of myeloid cells in HCC. Intratumor heterogeneity may result from the evolution of tumor cells and their continuous interactions with other cell types in the TME, which collectively drive tumorigenesis (Ma et al. 2022). CD74^+^ stromal macrophages impact the TME of HCC by increasing the infiltration of CD8^+^ CTLs (Xiao et al. 2022). Herein, we found that CD74 was widely expressed in macrophages, DCs, malignant cells and HPCs in HCC patients, which suggested a complex regulatory function of CD74 in the TME. The activation of the Hippo pathway in HPCs is essential for HCC development (Meng et al. 2021). High expression of the zinc finger protein ZEB1 promotes the differentiation of tumor-associated macrophages into cancer-promoting macrophages (M2) by upregulating CD74 expression in tumor cells, thus inducing immune escape and leading to poor prognosis (Cortes et al. 2017). Our findings revealed complex regulatory networks among multiple cell types during HCC development, especially between tumor cells and macrophages or between HPCs and DCs. CLEC9A(+) DCs are of major importance in the induction of antiviral and antitumor immunity (Aa et al. 2015). In this study, we found that high CD74 concentration in HPCs was associated with a decreased proportion of CLEC9A^(+)^ DCs, which suggested a potential tumor-promoting role of CD74 in HPCs. Additionally, high infiltration of IL-1B-positive macrophages was observed in abdominal aortic aneurysms (Cheng et al. 2022). In RCC, IL-1B-expressing macrophages are colocalized with high levels of epithelial–mesenchymal transition (EMT^high^) RCC cells macroscopically and microscopically both at the tumor-normal interface and in the tumor core, leading to tumor growth (Li et al. 2022a, b). Our data revealed a decrease in the number of IL-1B-positive macrophages among malignant CD74-overexpressing cells, which suggested that CD74 serves an antitumor role in malignant cells. CD74 expression in HPCs might exert carcinogenic effects, but high expression of CD74 in malignant cells may exert stronger antitumor effects.

In the TME, blocking the CD74-MIF axis restores the antitumor activity of macrophages and dendritic cells against melanoma (Figueiredo et al. 2018a, b). In hypersplenism resulting from HCC, B cells exert antitumor effects on a variety of cells through CD74-COPA (Zhao et al. 2022). Memory B cells in HCC exhibit high proliferation, low differentiation, and low activity, which are induced by the activated MIF-CD74 axis (Bai et al. 2022). CD74-MIF is expressed in the TME of many types of cancer after chemotherapy (Song et al. 2022). Reprogrammed TAMs affect the TME, and the cellular interaction between macrophages and malignant plasma cells is mediated by CD74-MIF, which reshapes the phenotypes of macrophages (Li et al. 2023). The crosstalk among CD8^+^ T cells, NKT cells, myeloid cells and cytotoxic plasma cells identified the potential immunotarget CD74-MIF (Zhong et al. 2022). In this study, both the interaction between IL-1B-positive macrophages and malignant cells and the interaction between CLEC9A-cDC1-positive DCs and malignant cells were evaluated mainly via the CD74-MIF axis. Therefore, the enriched ligand-receptor pair of CD74-MIF mainly mediated the crosstalk among immune cells and tumor cells in the TME of HCC, possibly through activating macrophages and other immune cells, including B cells, CD8^+^ T cells and NKT cells, in the TME.

TMB is related to immunotherapy efficacy in patients with HCC, as better tumor remission and clinical benefits were obtained from immunotherapy in patients with a higher TMB (Melendez et al. 2018). Neoantigens derived from nonsynonymous mutations in malignant tumor cells can be specifically recognized by T cells to elicit strong antitumor immune responses (Cai et al. 2021). There was no association between CD74 expression and TMB or neoantigen load in LIHC, indicating that the regulatory effect of CD74 on the TME is independent of TMB and neoantigen load. A high immune score (IS) predicts a better prognosis in pancreatic cancer patients than in patients with a low IS (Tahkola et al. 2018). According to our data, HCC patients with high CD74 expression had greater IS than patients with low CD74 expression, as reflected by the high stromal fraction, proliferation and wound healing activities and macrophage regulation. In tumors, a higher aneuploidy score is associated with poor prognosis after immunotherapy (Spurr et al. 2022). In CD74^high^ HCC patients, a lower aneuploidy score was observed than that in CD74^low^ patients, which further indicated that CD74 could positively affect prognosis after immunotherapy in HCC patients. However, the signaling network that mediates the interaction between IL-1B^+^ macrophages and malignant cells should be further studied using in vitro and in vivo experiments. The different roles of CD74 in immune cells and tumor cells should also be further explored in an in-depth way. Additionally, more clinical data should be collected and analyzed to prove the predictive effects of CD74 in HCC patients who undergo immunotherapy.

## Conclusion

In summary, CD74 was highly expressed in HCC samples and was significantly associated with alcohol abuse and hepatitis virus, which determine the prognosis of patients with HCC. Mechanistically, CD74 can shape the tumor microenvironment by regulating the development of myeloid cells, possibly by activating the innate immune response, such as interactions among HPCs, malignant cells, macrophages and DCs, thus facilitating immunotherapy for HCC. It is possible that targeting the CD74/MIF signaling axis in the TME may be a promising therapeutic strategy for HCC.

**Supplementary Figure legends**.

### Electronic supplementary material

Below is the link to the electronic supplementary material.

Supplementary Material 1

Supplementary Material 2

Supplementary Material 3

Supplementary Material 4

Supplementary Material 5

Supplementary Material 6

## Data Availability

All data generated or analyzed during this study are included in this published article and its additional files.

## Abbreviations

HCC: Hepatocellular carcinoma
DCs: dendritic cells
HPC: hepatic progenitor cells
TME: tumor microenvironment
NK: natural killer
MHC-II: major histocompatibility complex II
MIF: macrophage migratory inhibitory factor
CTLs: ytotoxic T lymphocytes
ENCORI: Encyclopedia of RNA Interactomes
TIMER: Tumor Immune Estimation Resource
HPA: Human Protein Atlas
KM: Kaplan-Meier
TISIDB: tumor-immune system interactions database
GEPIA2: Gene Expression Profiling Interactive Analysis 2
LinkedOmics: CAMOIP, Comprehensive Analysis on Multi-Omics of Immunotherapy in Pan-cancer
TIGER: Tissue-specific Gene Expression and Regulation
SCTIME: Single cell transcriptomes of tumor immune microenvironment
TISCH2: Tumor Immune Single Cell Hub 2
ROC: receiver operator characteristic
OS: overall survival
IHC: Immumohistochemical staining
CNV: copy number variation
LIHC: liver hepatocellular carcinoma
HBV/HCV: hepatitis B/C virus
DEGs: differentially expressed genes
TMB: Tumor mutational burden

## References

[CR40] Bai Y, Chen D, Cheng C, Li Z, Chi H, Zhang Y, et al. Immunosuppressive landscape in hepatocellular carcinoma revealed by single-cell sequencing. Front Immunol. 2022;13:950536. 35967424 10.3389/fimmu.2022.950536 PMC9365996

[CR3] Balkwill FR, Capasso M, Hagemann T. The tumor microenvironment at a glance. J Cell Sci. 2012;125:5591–6. 23420197 10.1242/jcs.116392

[CR4] Binnewies M, Roberts EW, Kersten K, Chan V, Fearon DF, Merad M, et al. Understanding the tumor immune microenvironment (TIME) for effective therapy. Nat Med. 2018;24:541–50. 29686425 10.1038/s41591-018-0014-x PMC5998822

[CR14] Borghese F, Clanchy FIL. CD74: an emerging opportunity as a therapeutic target in cancer and autoimmune disease. Expert Opin Ther Tar. 2011a;15:237–51. 10.1517/14728222.2011.550879 21208136

[CR28] Borghese F, Clanchy FI. CD74: an emerging opportunity as a therapeutic target in cancer and autoimmune disease. Expert Opin Ther Targets. 2011b;15:237–51. 21208136 10.1517/14728222.2011.550879

[CR45] Cai Z, Su X, Qiu L, Li Z, Li X, Dong X, et al. Personalized neoantigen vaccine prevents postoperative recurrence in hepatocellular carcinoma patients with vascular invasion. Mol Cancer. 2021;20:164. 34903219 10.1186/s12943-021-01467-8 PMC8667400

[CR24] Chen Z, Zhou L, Liu L, Hou Y, Xiong M, Yang Y, et al. Single-cell RNA sequencing highlights the role of inflammatory cancer-associated fibroblasts in bladder urothelial carcinoma. Nat Commun. 2020;11:5077. 33033240 10.1038/s41467-020-18916-5 PMC7545162

[CR15] Cheng SP, Liu CL, Chen MJ, Chien MN, Leung CH, Lin CH, et al. CD74 expression and its therapeutic potential in thyroid carcinoma. Endocr-Relat Cancer. 2015;22:179–90. 25600560 10.1530/ERC-14-0269

[CR37] Cheng S, Liu Y, Jing Y, Jiang B, Wang D, Chu X, et al. Identification of key monocytes/macrophages related gene set of the early-stage abdominal aortic aneurysm by integrated bioinformatics analysis and experimental validation. Front Cardiovasc Med. 2022;9:950961. 36186997 10.3389/fcvm.2022.950961 PMC9515382

[CR35] Cortes M, Sanchez-Moral L, de Barrios O, Fernandez-Acenero MJ, Martinez-Campanario MC, Esteve-Codina A, et al. Tumor-associated macrophages (TAMs) depend on ZEB1 for their cancer-promoting roles. Embo J. 2017;36:3336–55. 29038174 10.15252/embj.201797345 PMC5686549

[CR29] Cresswell P. Assembly, transport, and function of MHC class II molecules. Annu Rev Immunol. 1994;12:259–93. 8011283 10.1146/annurev.iy.12.040194.001355

[CR31] Du Y, Cai Y, Lv Y, Zhang L, Yang H, Liu Q, et al. Single-cell RNA sequencing unveils the communications between malignant T and myeloid cells contributing to tumor growth and immunosuppression in cutaneous T-cell lymphoma. Cancer Lett. 2022;551:215972. 36265653 10.1016/j.canlet.2022.215972

[CR18] Figueiredo CR, Azevedo RA, Mousdell S, Resende-Lara PT, Ireland L, Santos A et al. Blockade of MIF-CD74 Signalling on macrophages and dendritic cells restores the Antitumour Immune Response against Metastatic Melanoma. Front Immunol 2018a;9. 10.3389/fimmu.2018.01132 PMC5974174 29875777

[CR38] Figueiredo CR, Azevedo RA, Mousdell S, Resende-Lara PT, Ireland L, Santos A, et al. Blockade of MIF-CD74 Signalling on macrophages and dendritic cells restores the Antitumour Immune Response against Metastatic Melanoma. Front Immunol. 2018b;9:1132. 29875777 10.3389/fimmu.2018.01132 PMC5974174

[CR23] Gai JW, Wahafu W, Song LM, Ping H, Wang MS, Yang FY, et al. Expression of CD74 in bladder cancer and its suppression in association with cancer proliferation, invasion and angiogenesis in HT-1376 cells. Oncol Lett. 2018;15:7631–8. 29731899 10.3892/ol.2018.8309 PMC5920967

[CR19] Ghoochani A, Schwarz MA, Yakubov E, Engelhorn T, Doerfler A, Buchfelder M, et al. MIF-CD74 signaling impedes microglial M1 polarization and facilitates brain tumorigenesis. Oncogene. 2016;35:6246–61. 27157615 10.1038/onc.2016.160

[CR7] Lei Y, Tang R, Xu J, Wang W, Zhang B, Liu J, et al. Applications of single-cell sequencing in cancer research: progress and perspectives. J Hematol Oncol. 2021;14:91. 34108022 10.1186/s13045-021-01105-2 PMC8190846

[CR13] Li R, Ferdinand JR, Loudon KW, Bowyer GS, Laidlaw S, Muyas F, et al. Mapping single-cell transcriptomes in the intra-tumoral and associated territories of kidney cancer. Cancer Cell. 2022a;40:1583–99. e1510. 36423636 10.1016/j.ccell.2022.11.001 PMC9767677

[CR26] Li XY, Shen Y, Zhang L, Guo X, Wu J. Understanding initiation and progression of hepatocellular carcinoma through single cell sequencing. Biochim Biophys Acta Rev Cancer. 2022b;1877:188720. 35304295 10.1016/j.bbcan.2022.188720

[CR42] Li J, Yang Y, Wang W, Xu J, Sun Y, Jiang J, et al. Single-cell atlas of the immune microenvironment reveals macrophage reprogramming and the potential dual macrophage-targeted strategy in multiple myeloma. Br J Haematol. 2023;201:917–34. 36852636 10.1111/bjh.18708

[CR34] Ma L, Heinrich S, Wang L, Keggenhoff FL, Khatib S, Forgues M, et al. Multiregional single-cell dissection of tumor and immune cells reveals stable lock-and-key features in liver cancer. Nat Commun. 2022;13:7533. 36476645 10.1038/s41467-022-35291-5 PMC9729309

[CR8] Mao X, Xu J, Wang W, Liang C, Hua J, Liu J, et al. Crosstalk between cancer-associated fibroblasts and immune cells in the tumor microenvironment: new findings and future perspectives. Mol Cancer. 2021;20:131. 34635121 10.1186/s12943-021-01428-1 PMC8504100

[CR44] Melendez B, Van Campenhout C, Rorive S, Remmelink M, Salmon I, D’Haene N. Methods of measurement for tumor mutational burden in tumor tissue. Transl Lung Cancer Res. 2018;7:661–7. 30505710 10.21037/tlcr.2018.08.02 PMC6249625

[CR12] Meng Y, Zhao Q, An L, Jiao S, Li R, Sang Y, et al. A TNFR2-hnRNPK Axis promotes primary Liver Cancer Development via activation of YAP Signaling in hepatic progenitor cells. Cancer Res. 2021;81:3036–50. 33619115 10.1158/0008-5472.CAN-20-3175

[CR21] Meyer-Siegler KL, Iczkowski KA, Leng L, Bucala R, Vera PL. Inhibition of macrophage migration inhibitory factor or its receptor (CD74) attenuates growth and invasion of DU-145 prostate cancer cells. J Immunol. 2006;177:8730–9. 17142775 10.4049/jimmunol.177.12.8730

[CR1] Murai H, Kodama T, Maesaka K, Tange S, Motooka D, Suzuki Y, et al. Multiomics identifies the link between intratumor steatosis and the exhausted tumor immune microenvironment in hepatocellular carcinoma. Hepatology. 2023;77:77–91. 35567547 10.1002/hep.32573 PMC9970024

[CR6] Peneau C, Imbeaud S, La Bella T, Hirsch TZ, Caruso S, Calderaro J, et al. Hepatitis B virus integrations promote local and distant oncogenic driver alterations in hepatocellular carcinoma. Gut. 2022;71:616–26. 33563643 10.1136/gutjnl-2020-323153 PMC8862055

[CR5] Shen M, Kang Y. Complex interplay between tumor microenvironment and cancer therapy. Front Med. 2018;12:426–39. 30097962 10.1007/s11684-018-0663-7

[CR2] Siegel RL, Miller KD, Wagle NS, Jemal A. Cancer statistics, 2023. CA Cancer J Clin. 2023;73:17–48. 36633525 10.3322/caac.21763

[CR41] Song H, Lou C, Ma J, Gong Q, Tian Z, You Y, et al. Single-cell transcriptome analysis reveals changes of Tumor Immune Microenvironment in oral squamous cell Carcinoma after Chemotherapy. Front Cell Dev Biol. 2022;10:914120. 35784460 10.3389/fcell.2022.914120 PMC9247458

[CR47] Spurr LF, Weichselbaum RR, Pitroda SP. Tumor aneuploidy predicts survival following immunotherapy across multiple cancers. Nat Genet. 2022;54:1782–5. 36443443 10.1038/s41588-022-01235-4

[CR22] Ssadh HA, Abdulmonem WA, Rasheed Z, Madar IH, Alhoderi J, Eldeen SKN, et al. Knockdown of CD-74 in the proliferative and apoptotic activity of breast Cancer cells. Open Access Maced J Med Sci. 2019;7:3169–76. 31949511 10.3889/oamjms.2019.354 PMC6953917

[CR33] Su H, Na N, Zhang X, Zhao Y. The biological function and significance of CD74 in immune diseases. Inflamm Res. 2017;66:209–16. 27752708 10.1007/s00011-016-0995-1

[CR46] Tahkola K, Mecklin JP, Wirta EV, Ahtiainen M, Helminen O, Bohm J, et al. High immune cell score predicts improved survival in pancreatic cancer. Virchows Arch. 2018;472:653–65. 29356891 10.1007/s00428-018-2297-1

[CR36] van der Aa E, van Montfoort N, Woltman AM. BDCA3(+)CLEC9A(+) human dendritic cell function and development. Semin Cell Dev Biol. 2015;41:39–48. 24910448 10.1016/j.semcdb.2014.05.016

[CR17] Wang ZQ, Milne K, Webb JR, Watson PH. CD74 and intratumoral immune response in breast cancer. Oncotarget. 2017;8:12664–74. 27058619 10.18632/oncotarget.8610 PMC5355043

[CR10] Wang S, Wu Q, Chen T, Su R, Pan C, Qian J, et al. Blocking CD47 promotes antitumour immunity through CD103(+) dendritic cell-NK cell axis in murine hepatocellular carcinoma model. J Hepatol. 2022;77:467–78. 35367532 10.1016/j.jhep.2022.03.011

[CR30] Wang R, Peng L, Xiao Y, Zhou Q, Wang Z, Tang L, et al. Single-cell RNA sequencing reveals changes in glioma-associated macrophage polarization and cellular states of malignant gliomas with high AQP4 expression. Cancer Gene Ther. 2023;30:716–26. 36599974 10.1038/s41417-022-00582-y PMC10191842

[CR25] Wirtz TH, Saal A, Bergmann I, Fischer P, Heinrichs D, Brandt EF, et al. Macrophage migration inhibitory factor exerts pro-proliferative and anti-apoptotic effects via CD74 in murine hepatocellular carcinoma. Br J Pharmacol. 2021;178:4452–67. 34250589 10.1111/bph.15622

[CR20] Xiao N, Li K, Zhu X, Xu B, Liu X, Lei M, et al. CD74(+) macrophages are associated with favorable prognosis and immune contexture in hepatocellular carcinoma. Cancer Immunol Immunother. 2022;71:57–69. 34009409 10.1007/s00262-021-02962-z PMC10992586

[CR9] Yang P, Qin H, Li Y, Xiao A, Zheng E, Zeng H, et al. CD36-mediated metabolic crosstalk between tumor cells and macrophages affects liver metastasis. Nat Commun. 2022;13:5782. 36184646 10.1038/s41467-022-33349-y PMC9527239

[CR11] Zeng FL, Wang XY, Hu YW, Wang Z, Li Y, Hu J, et al. Interleukin-37 promotes DMBA/TPA skin cancer through SIGIRR-mediated inhibition of glycolysis in CD103(+)DC cells. MedComm (2020). 2023;4:e229. 36891351 10.1002/mco2.229 PMC9986080

[CR27] Zhang JF, Tao LY, Yang MW, Xu DP, Jiang SH, Fu XL, et al. CD74 promotes perineural invasion of cancer cells and mediates neuroplasticity via the AKT/EGR-1/GDNF axis in pancreatic ductal adenocarcinoma. Cancer Lett. 2021;508:47–58. 33766751 10.1016/j.canlet.2021.03.016

[CR16] Zhao SC, Molina A, Yu A, Hanson J, Cheung H, Li XF, et al. High frequency of CD74 expression in lymphomas: implications for targeted therapy using a novel anti-CD74-drug conjugate. J Pathol Clin Res. 2019;5:12–24. 30191677 10.1002/cjp2.114 PMC6317062

[CR39] Zhao HC, Chen CZ, Song HQ, Wang XX, Zhang L, Zhao HL, et al. Single-cell RNA sequencing analysis reveals New Immune disorder complexities in Hypersplenism. Front Immunol. 2022;13:921900. 35865544 10.3389/fimmu.2022.921900 PMC9294158

[CR43] Zhong L, Hao P, Zhang Q, Jiang T, Li H, Xiao J et al. Revised International Staging System (R-ISS) stage-dependent analysis uncovers oncogenes and potential immunotherapeutic targets in multiple myeloma (MM). Elife 2022;11. 10.7554/eLife.75340 PMC9674343 36315425

[CR32] Zhong G, Lin Y, Huang Z. Identification of a novel circRNA-miRNA-mRNA regulatory axis in hepatocellular carcinoma based on bioinformatics analysis. Sci Rep. 2023;13:3728. 36878930 10.1038/s41598-023-30567-2 PMC9988886

