# Supplementary figures and images for: CD74 facilitates immunotherapy response by shaping the tumor microenvironment of hepatocellular carcinoma
Source: Mol Med. 2024 Aug 8;30:116. doi: 10.1186/s10020-024-00884-x (PMC11308498; doi:10.1186/s10020-024-00884-x)

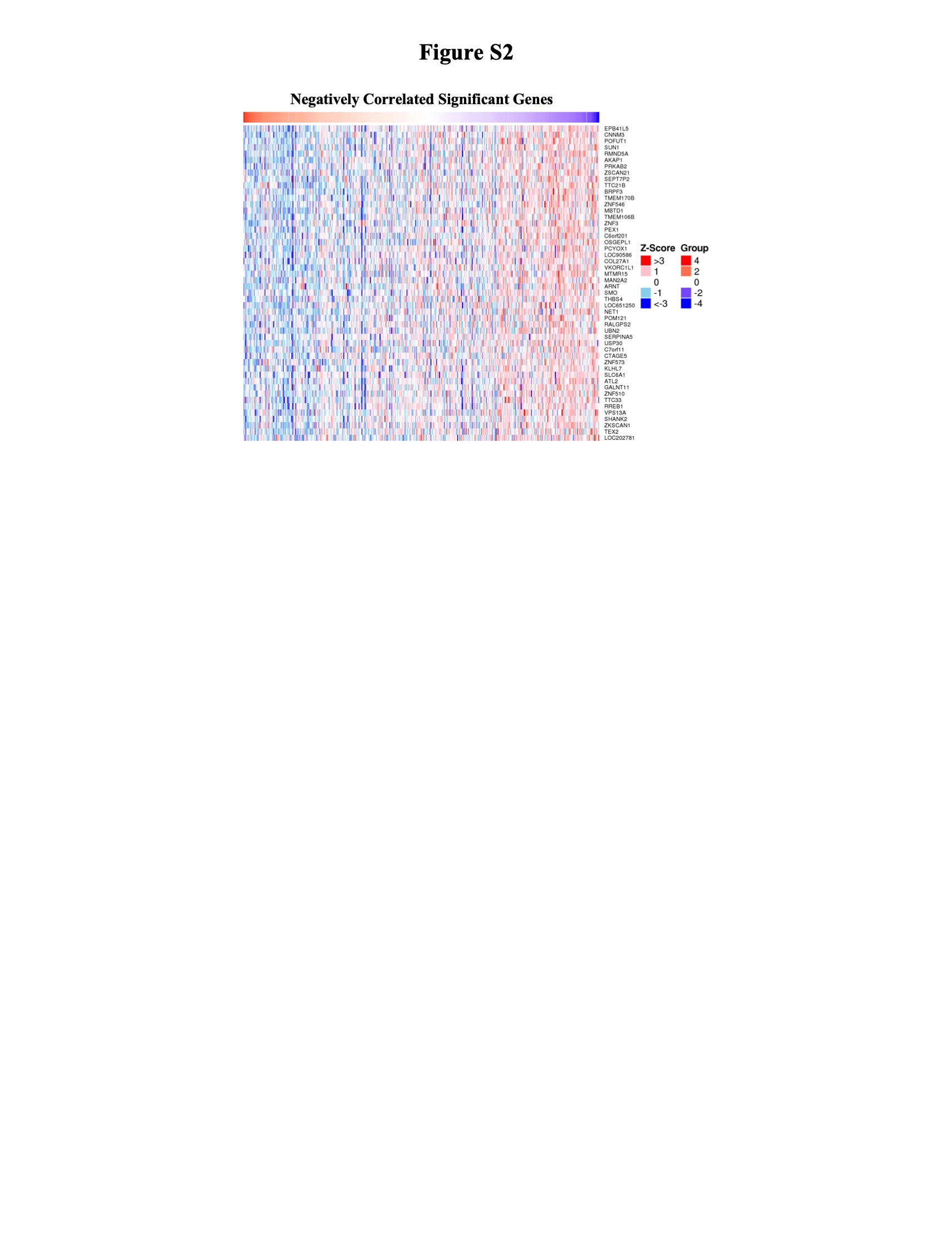

Supplement: Supplementary file 1 — Supplementary Material 1 [file 10020_2024_884_MOESM1_ESM.jpeg]

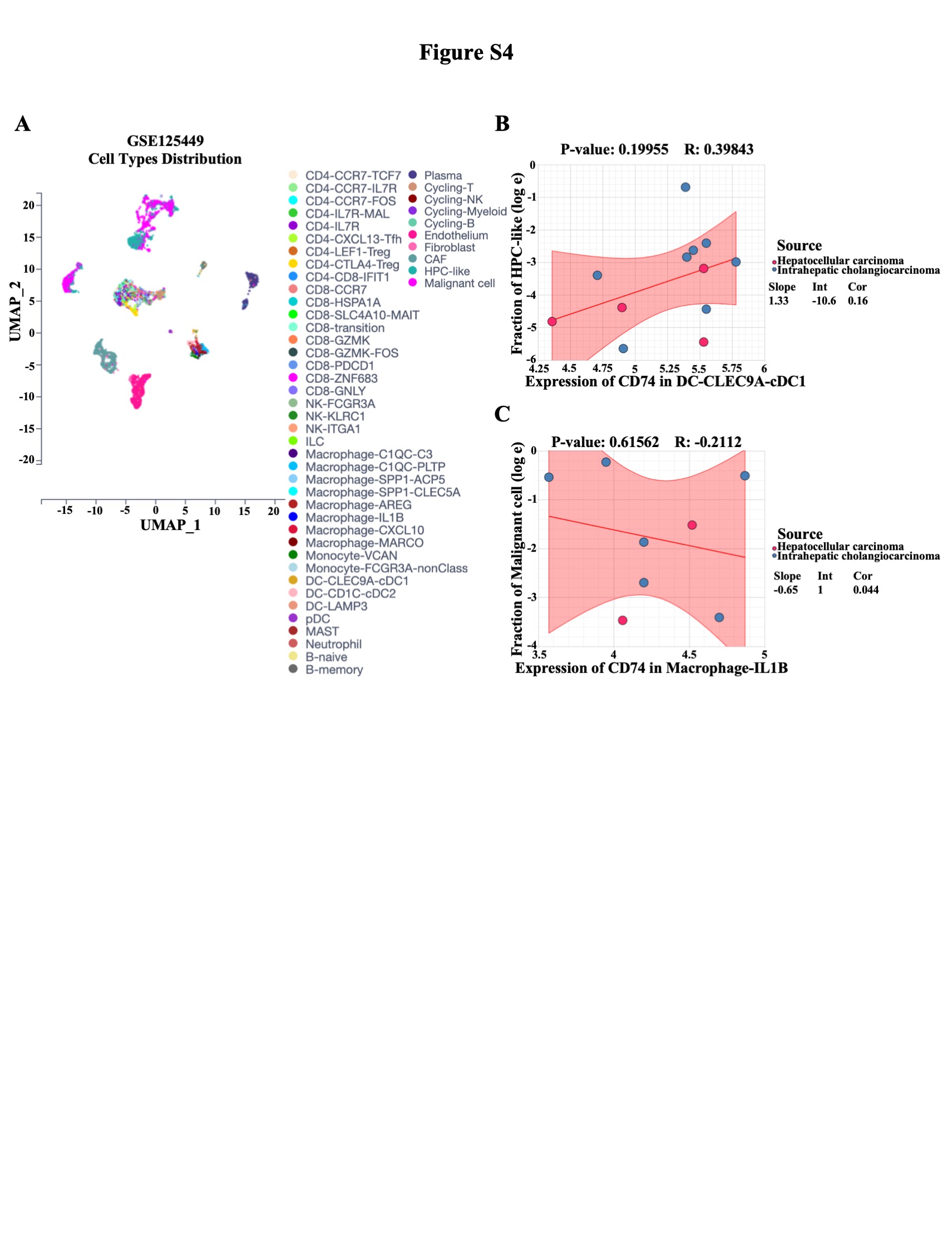

Supplement: Supplementary file 2 — Supplementary Material 2 [file 10020_2024_884_MOESM2_ESM.jpeg]

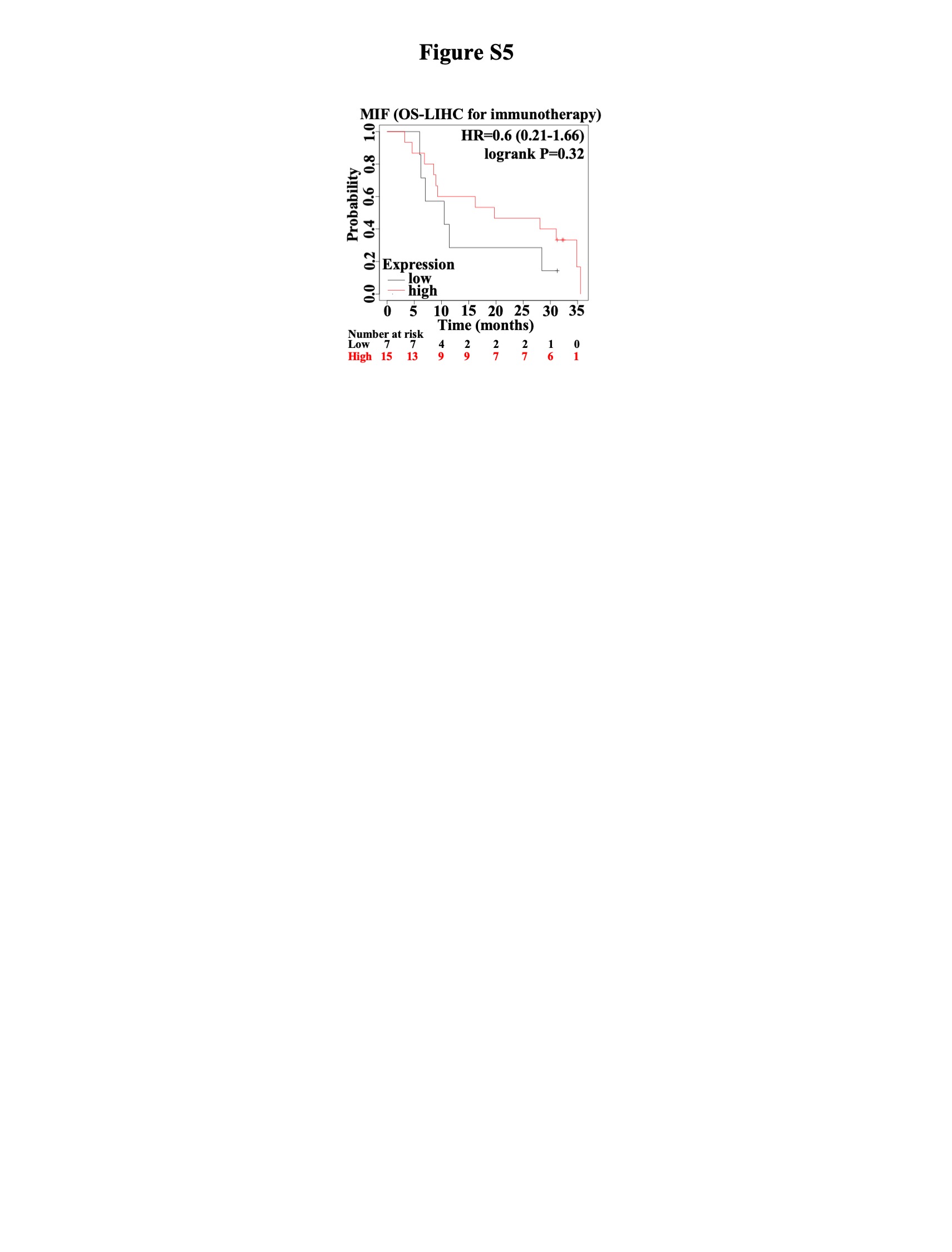

Supplement: Supplementary file 3 — Supplementary Material 3 [file 10020_2024_884_MOESM3_ESM.jpeg]

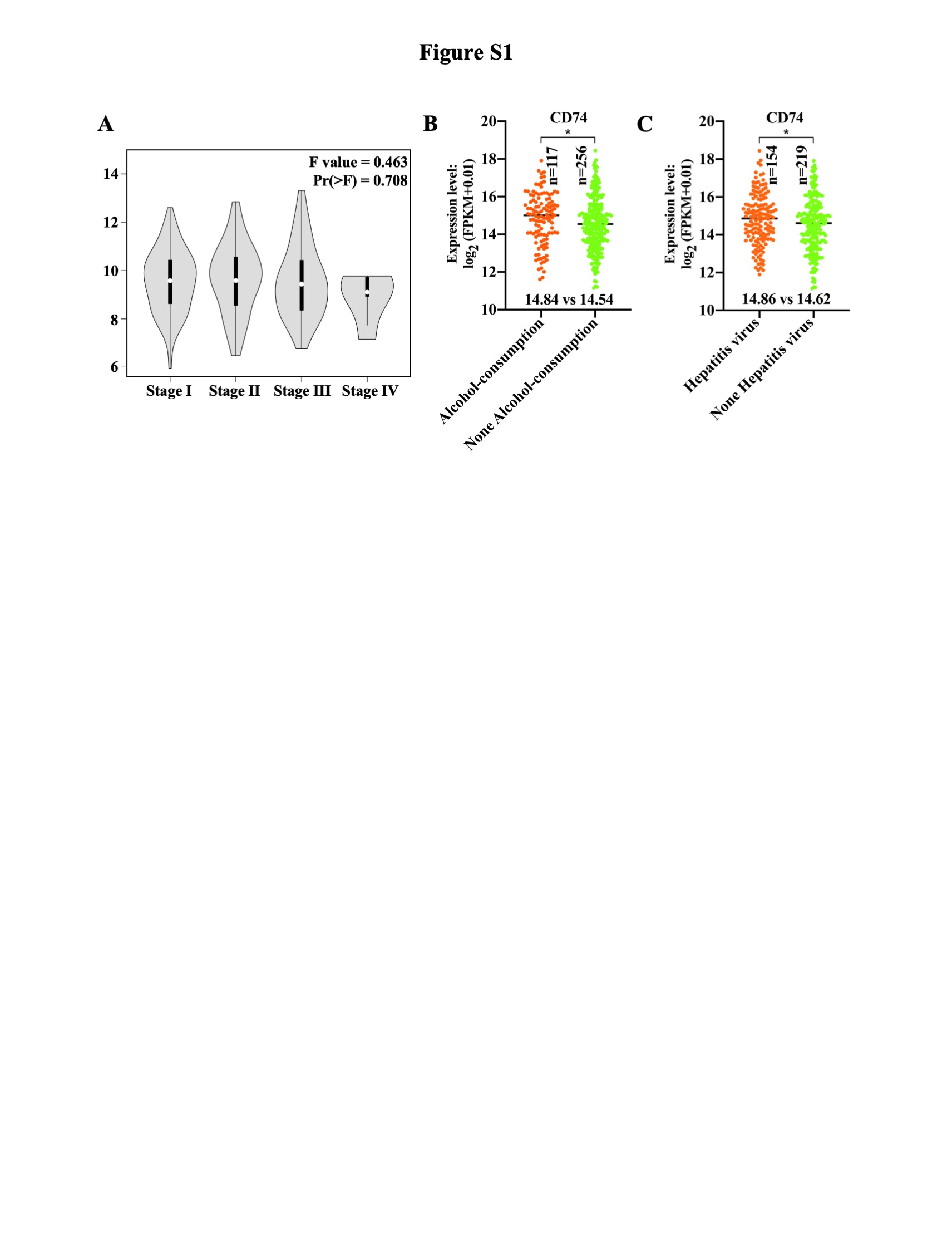

Supplement: Supplementary file 5 — Supplementary Material 5 [file 10020_2024_884_MOESM5_ESM.jpeg]

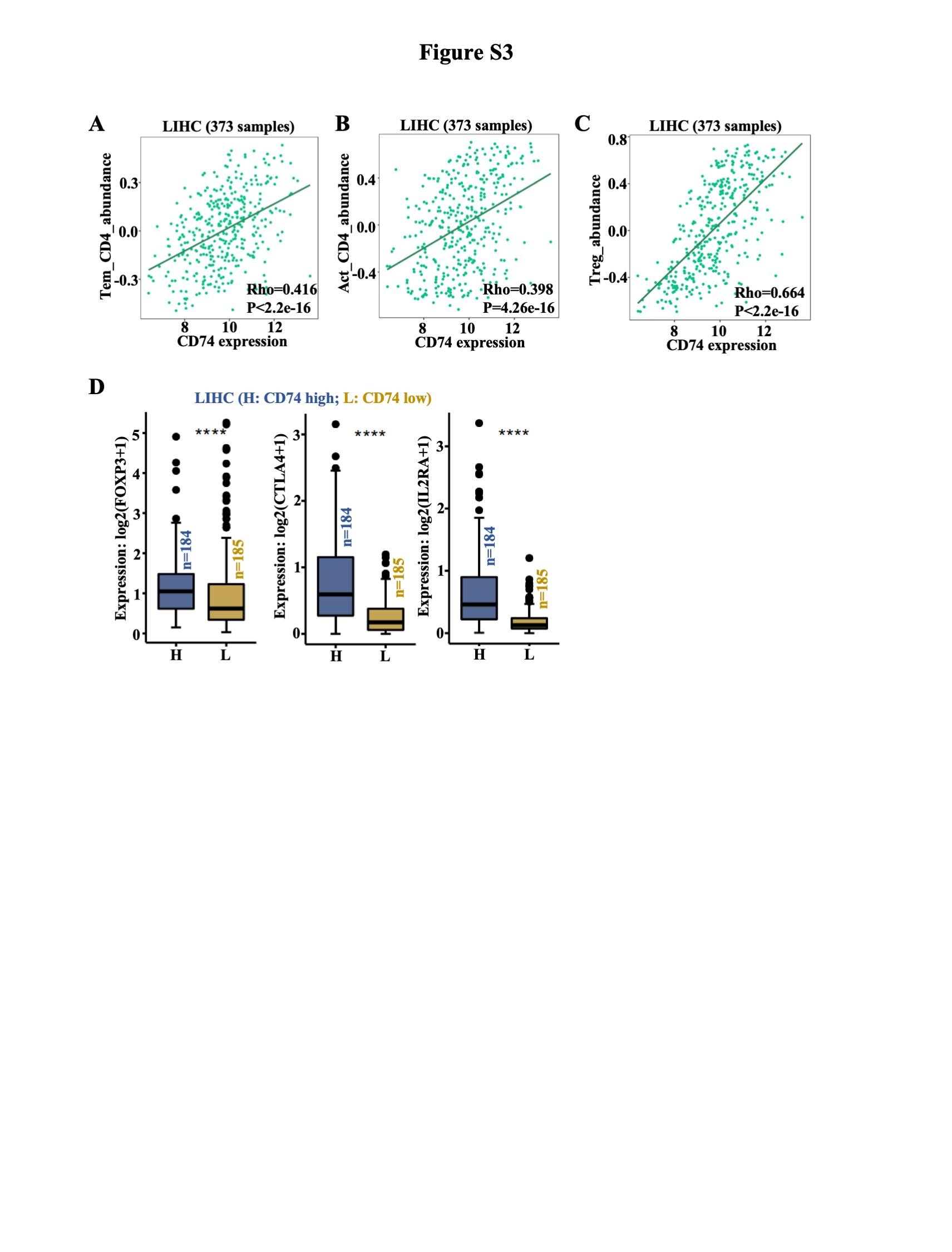

Supplement: Supplementary file 6 — Supplementary Material 6 [file 10020_2024_884_MOESM6_ESM.jpeg]
