# Supplementary material for: CD74 facilitates immunotherapy response by shaping the tumor microenvironment of hepatocellular carcinoma
Source: Mol Med. 2024 Aug 8;30:116. doi: 10.1186/s10020-024-00884-x (PMC11308498; doi:10.1186/s10020-024-00884-x)
Supplement: Supplementary file 4 — Supplementary Material 4 [file 10020_2024_884_MOESM4_ESM.docx]

**Supplementary Figure legends**

**Figure S1 Differential expression of CD74 in LIHC patients with different tumor stages and risk factors. (A)** Box plot showing the relative expression of CD74 in normal subjects or LIHC patients with stage I, II, III, or IV disease. **(B)** Relative expression of CD74 in normal subjects or LIHC patients who did or did not consume alcohol. **(C)** Relative expression of CD74 in normal controls or LIHC patients who were positive for hepatitis virus and who were negative for hepatitis virus. *, p < 0.05.

**Figure S2 Association analysis of genes coexpressed with CD74 in LIHC.** Heatmaps showing genes negatively associated with CD74 in LIHC (top 50). Red indicates a positive gene correlation, and green indicates a negative gene correlation.

**Figure S3** **Relationship between CD74 expression and immune cell abundance in LIHC.** **(A-C)** Association between CD74 expression and Tem-CD4^+^ T-cell abundance (A), Act-CD4^+^ T-cell abundance (B) and Treg abundance (C) in LIHC according to the TIMER database. **(D)** Gene expression levels of the activated Treg cell markers FOXP3, CTAL4 and IL-2RA in high CD74 (n=184) or low CD74 (n=185) liver tumor tissues according to the CAMOIP database. ****, p < 0.0001.

**Figure S4 Association analysis between cell composition and CD74 expression. (A)** UMAP plot showing the distributions of all cell types combined with single-cell data retrieved from the Gene Expression Omnibus (GSE125449). **(B)** Correlation analysis of the cell composition of hepatic progenitor cells (HPCs) and CD74 expression in CLEC9A-cDC1^+^ DCs. **(C)** Correlation analysis of the cell composition of malignant cells and CD74 expression in IL-1B^+^ macrophages.

**Figure S5 Overall survival curve of LIHC patients with differential MIF expression who received immunotherapy.**
